# Supplementary material for: The Perlman syndrome DIS3L2 exoribonuclease safeguards endoplasmic reticulum-targeted mRNA translation and calcium ion homeostasis
Source: Nat Commun. 2020 May 26;11:2619. doi: 10.1038/s41467-020-16418-y (PMC7250864; doi:10.1038/s41467-020-16418-y)
Supplement: Supplementary file 1 — Supplementary Information [file 41467_2020_16418_MOESM1_ESM.pdf]

Supplementary Information:

Pirouz et al., The Perlman syndrome DIS3L2 exoribonuclease safeguards endoplasmic reticulum-targeted mRNA translation and calcium ion homeostasis

Supplementary Figure 1

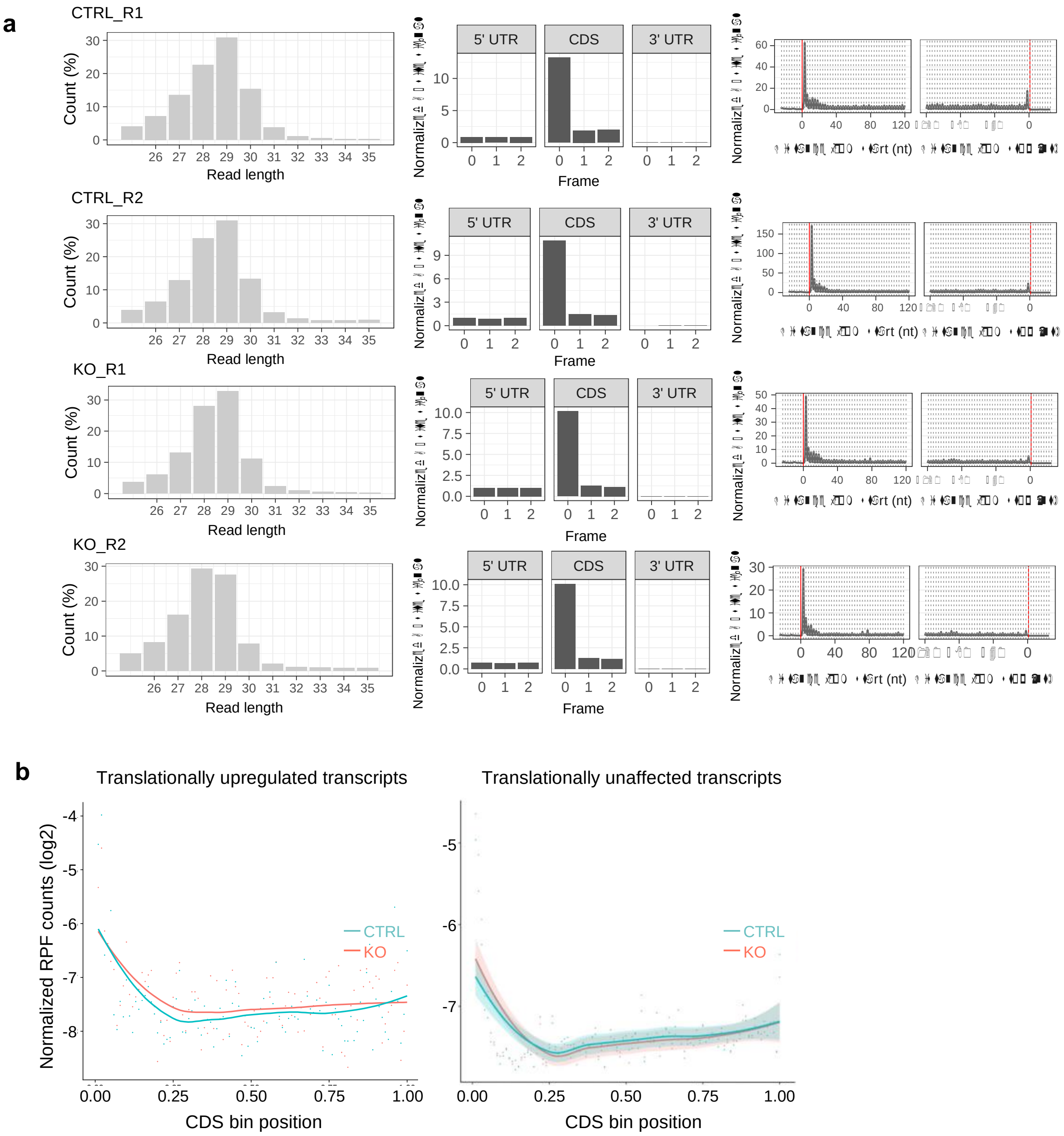

Supplementary Figure 1. Characteristics of ribosome profiling data in control and DIS3L2 knockout mESCs. a, Left panels: ribosome protected fragment (RPF) length distribution; middle panels: normalized A-site signals of RPFs for different coding frames among the 5’-UTR, coding sequence (CDS) and 3’-UTR in ribosome profiling data; right panels: normalized A-site signals of RPFs near the translation start sites (TSS) and translation end sites (TES). **b,** Overall distribution of RPFs among translationally upregulated (left panel) or unaffected (right panel) mRNAs.

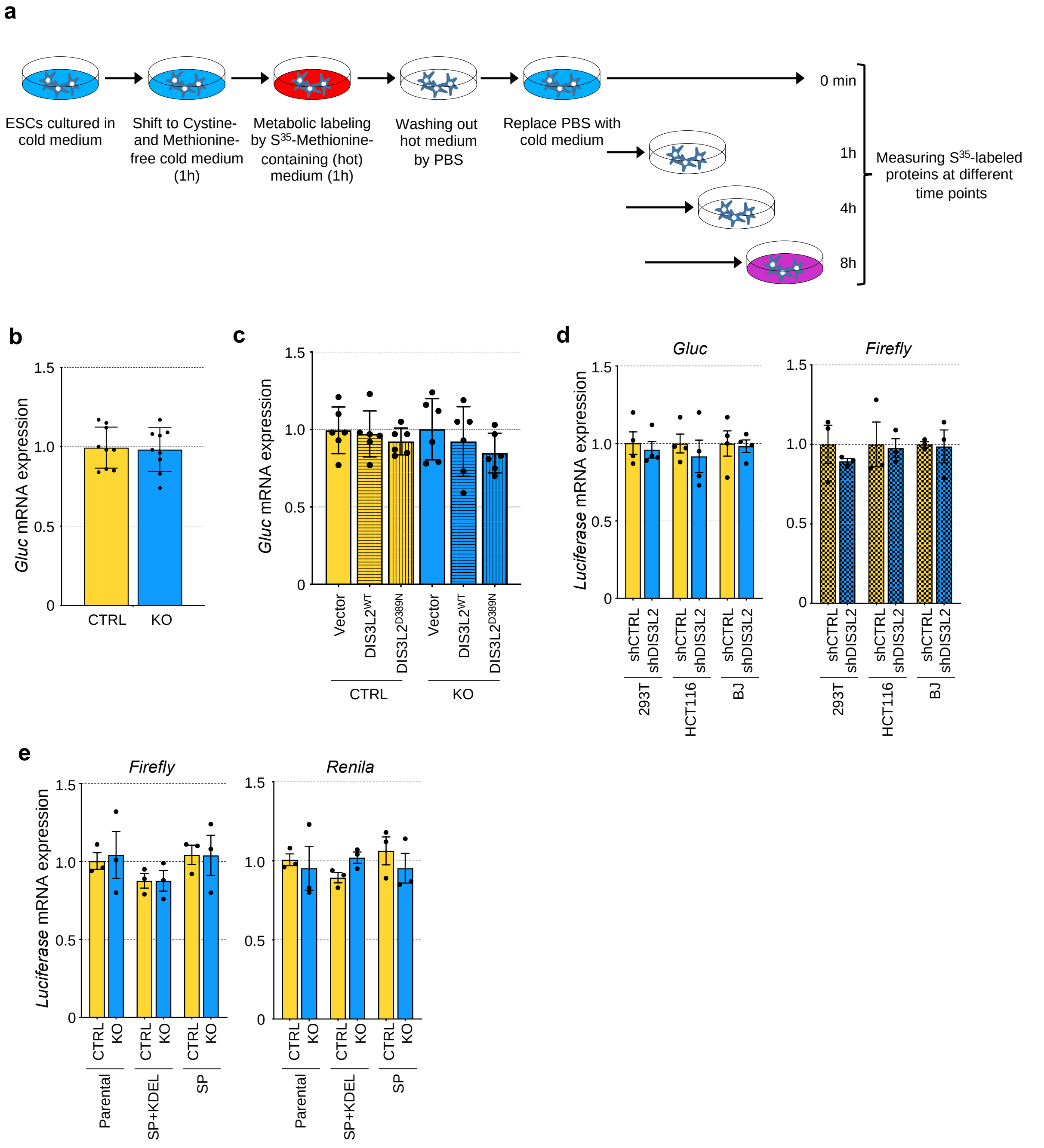

Supplementary Figure 2. Defective ER-targeted mRNA translation upon DIS3L2 depletion. a, A schematic representation of the metabolic labeling experiment in Figure 2a. Media containing radiolabeled S_{35} is marked in red. The gradient of purple color indicates increasing accumulation of S_{35}-labeled proteins secreted to the supernatant after replacing the PBS with cold medium (without S_{35}).

qRT-PCR analysis of indicated luciferase reporter mRNA expression is shown as for: **b,** Figure 2c (n=9 independent experiments). **c,** Figure 2d (n=6 independent experiments). **d,** Figure 2e (n≥3 independent experiments). **e,** Figure 2f (n=3 independent experiments). In **b-e**, bars represent mean ± SEM. Source data are provided as a Source Data.

Supplementary Figure 3

a

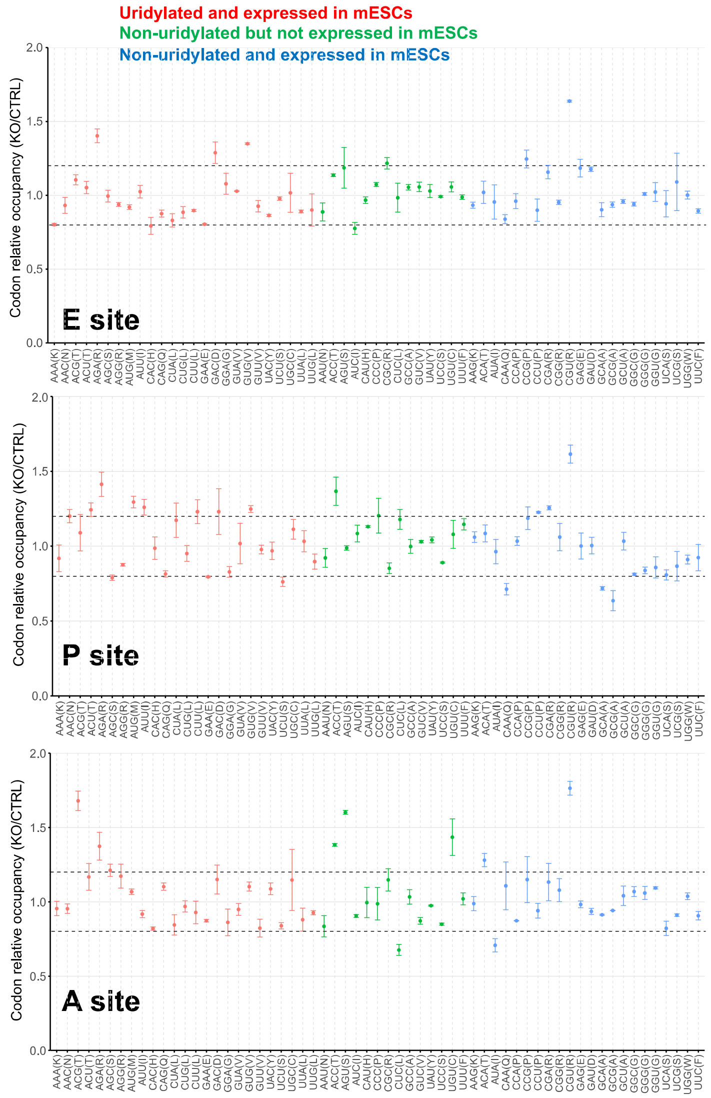

b

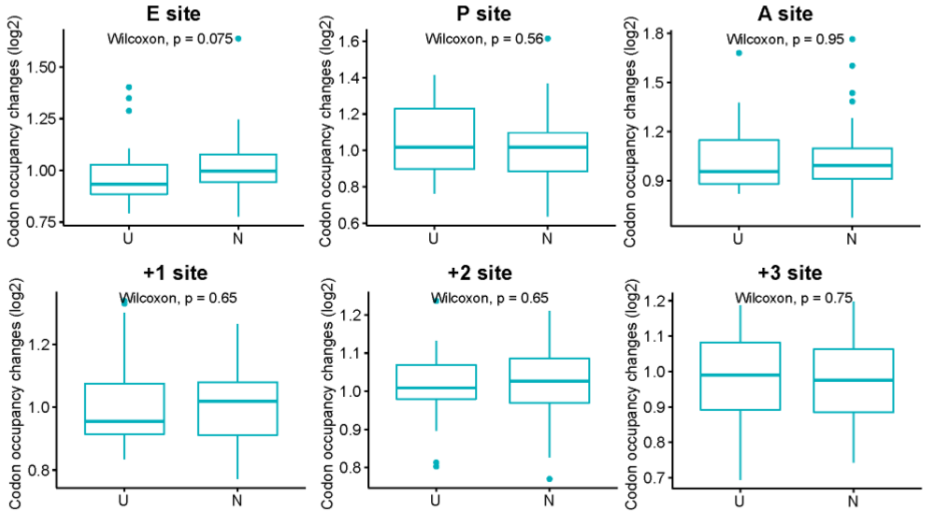

Supplementary Figure 3. Normal codon usage frequencies in DIS3L2 knockout mESCs. a, Relative codon occupancies changes at E-, P-, and A-sites in ribosome profiling data from control and DIS3L2 knockout ESCs. Codons related to DIS3L2-targeted tRNAs which were determined in other study[12] are marked as “uridylated”. The expression status of tRNAs in mESC were conducted in our previous study[79]. **b,** No significant changes were observed between codon occupancies related to DIS3L2-targeted tRNAs (U), and those that are not targeted by DIS3L2 (N) (Wilcoxon rank sum test). Box plots display the full range of variation on the basis of the five number summaries (minimum, first quartile, median, third quartile, and maximum). P values are from two-sided Wilcoxon signed-rank tests.

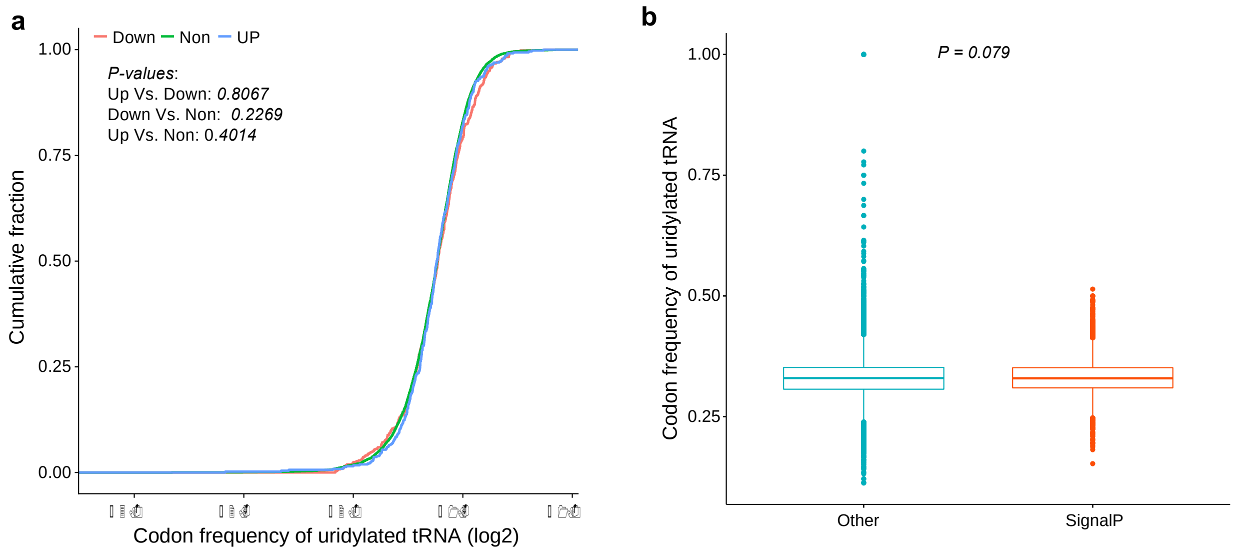

Supplementary Figure 4. Normal codon usage frequencies in DIS3L2 knockout mESCs. a, No significant changes were detected in codon frequencies of DIS3L2-targeted tRNAs among differentially translated (up or down) genes and Non-changed ones (P values are from two-sided Kolmogorov–Smirnov tests). **b,** No significant changes were detected in the codon frequencies of uridylated tRNAs among signal peptide-containing genes (SignalP) and rest of the expressed genes (Wilcoxon rank sum test). Box plots display the full range of variation on the basis of the five number summaries (minimum, first quartile, median, third quartile, and maximum). P value is from a two-sided Wilcoxon signed-rank test.

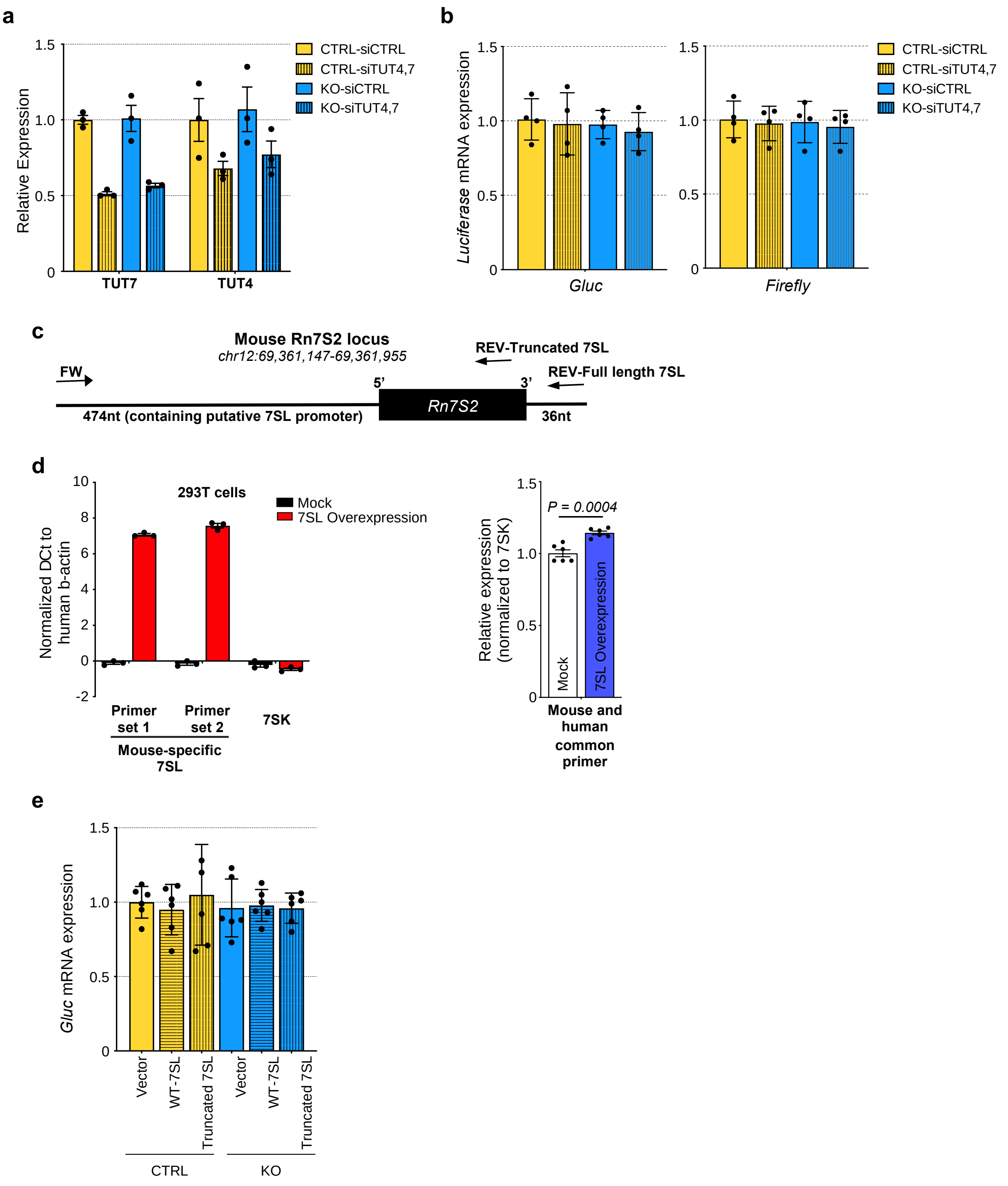

Supplementary Figure 5. DIS3L2-mediated quality control of 7SL. a, qRT-PCR analysis of TUTase depletion in control and DIS3L2 KO mESCs (n=3 independent experiments). **b,** qRT-PCR analysis of indicated luciferase mRNA expression related to Figure 3h (n=4 independent experiments). **c,**

Schematic representation of the 7SL RNA locus and the primer oligos used for amplification and cloning of the full-length WT and truncated 7SL RNA into pGEM-T easy vectors. **d,** Verification of ectopic expression of murine 7SL RNA in human HEK293T cells using mouse-specific (left) and common (right) primer sets (n=3 independent experiments). **e,** qRT-PCR analysis of indicated luciferase mRNA expression related to Figure 3i (n=6 independent experiments). Bars represent mean ± SEM. *P* values (unpaired 2-tailed Student's t-test, 0.95 confidence intervals) are shown. Source data are provided as a Source Data file.

Supplementary Figure 6

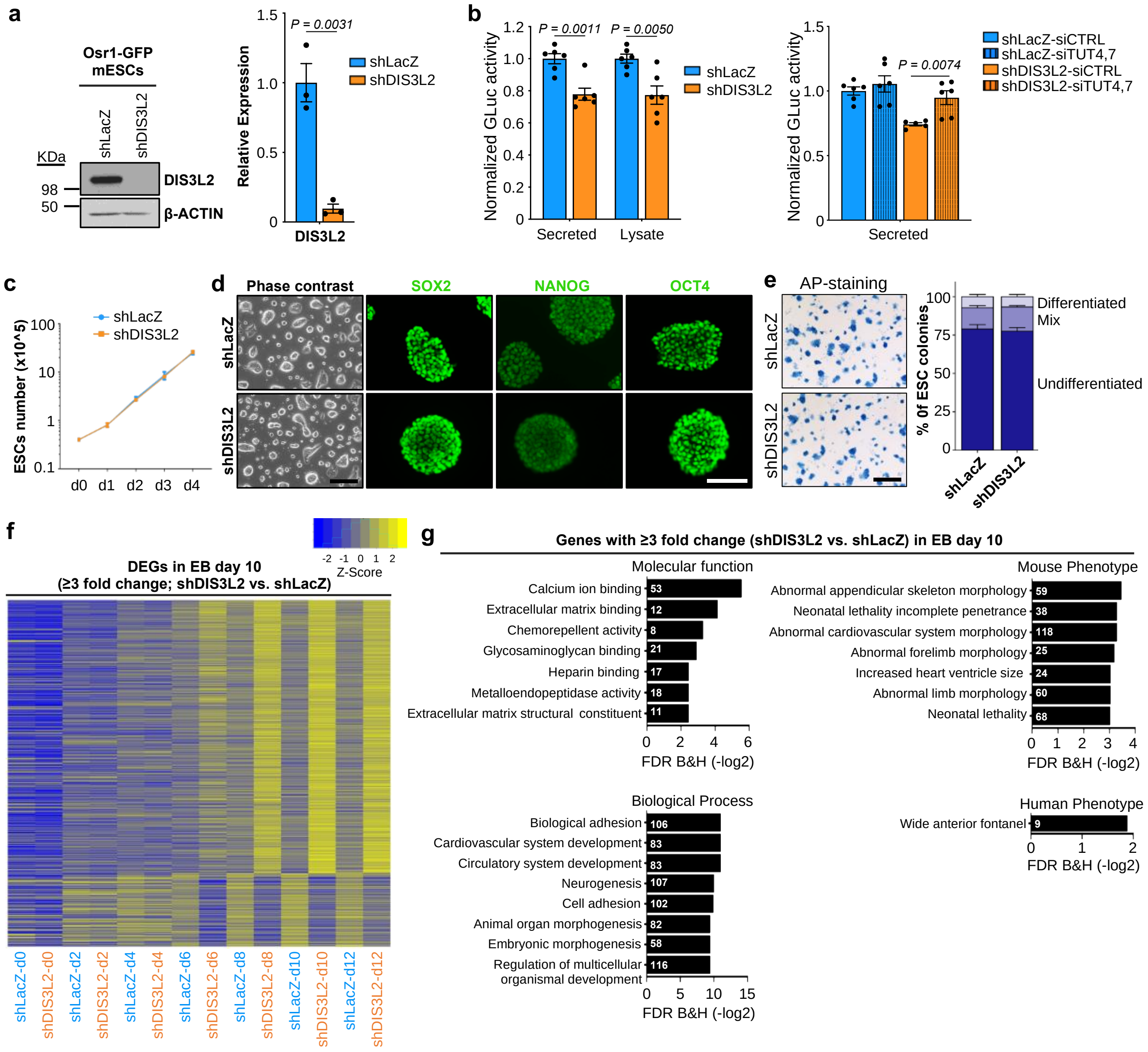

Supplementary Figure 6. Dysregulated differentiation of DIS3L2-depleted mESCs. a, Confirmation of DIS3L2 knockdown by western blot (left) and qRT-PCR (right) in Osr1-GFP reporter mESC line. Left panel: Representative images of three independent experiments with similar results are provided. Ricght panel: (n=3 independent experiments). **b,** Left panel: normalized activity of Gluc reporter in stable DIS3L2 knockdown mESCs. Right panel: the effect of TUTases depletion on Gluc reporter secretion in stable DIS3L2 knockdown mESCs (n=6 independent experiments). **c,** Cell proliferation of Osr1-GFP

reporter ESCs with stable DIS3L2, or LacZ depletion (n=3 biologically independent samples). **d,** Phase contrast images and immunofluorescent staining of pluripotency markers expression. Black scale bar = 200 μm; White scale bar = 100 μm. **e,** Alkaline Phosphatase (AP) staining and quantification of Osr1-GFP reporter ESC lines (n=4 non-overlapping fields were measured). Scale bar = 200 μm. In **d, e**, representative images of two independent experiments with similar results are provided. **f,** Heatmap representation of differentially expressed genes at d10 during the entire course of differentiation. **g,** Gene ontology analysis of DEGs from **(f)** at d10 of differentiation. numbers inside the bars indicate the number of DEGs associated with each biological terms. In **(a-c, e)**, bars representing mean ± SEM and *P* values (unpaired 2-tailed Student's t-test, 0.95 confidence intervals) are indicated. Source data are provided as a Source Data file.

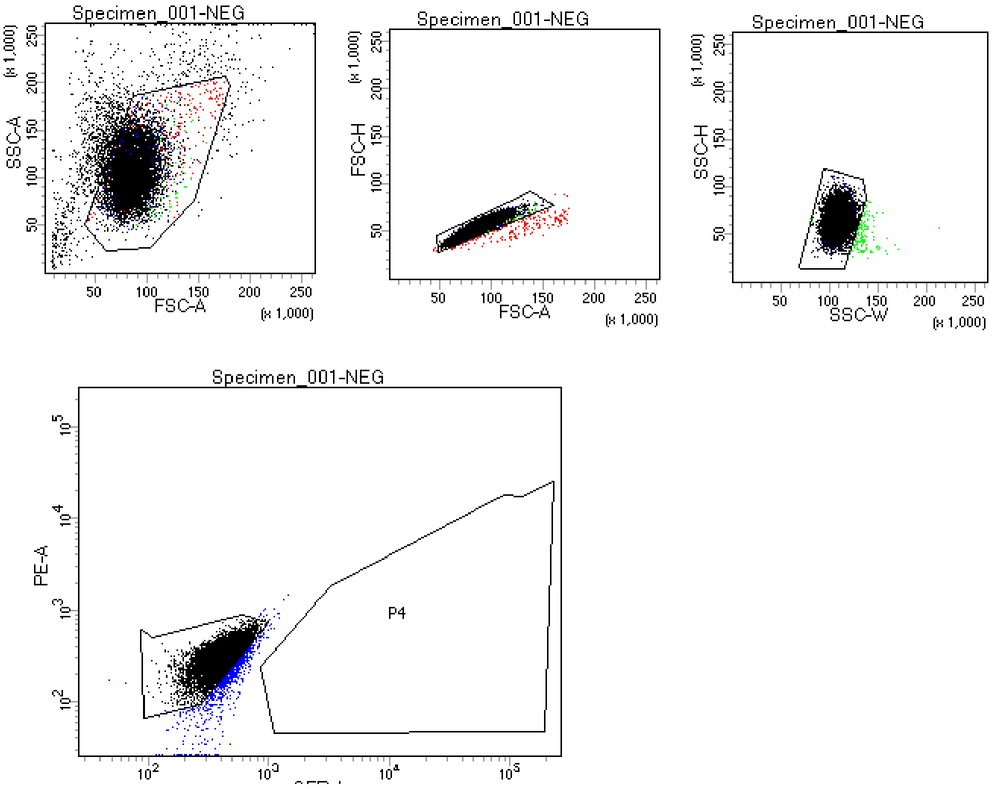

Supplementary Figure 7. Gating strategy in flow cytometry analysis of Osr1-GFP$_{\text{positive}}$ cells. Top panels: Debris and doublets were excluded based on forward and side scatter profiles. Bottom panel: GFP expression in negative control cells (undifferentiated ESCs).

Supplementary Table 1: List of primers and oligonucleotides

List of oligonucleotide primers and oligos

NAME	SEQUENCE 5'-3'	NOTE
**qRT-PCR primers**		
Renila-FW	CTGATCTGATCGGAATGGGTAAG	
Renila-REV	CAAGCGGTGAGGTACTTGTAG	
Firefly-FW	ACAAGGCCATGAAGAGGTATG	
Firefly-REV	GAAGTACTCGGCATAGGTGATG	
Gluc-FW	CACGCCCAAGATGAAGAAGT	
Gluc-REV	GAACCCAGGAATCTCAGGAATG	
Dis3l2-FW	GATCAGTGGAAGCCAAGGATAA	
Dis3l2-REV	CAGGGATGTCAGCTTCATAAG	
Six2-FW	TCTCTCTCTCTCTCTCTCTCTCT	
Six2-REV	GTCTACCTCCGTTCACATCAC	
LIM1-FW	TAGCCGTGAGTGAGGAAGTG	
LIM1-REV	CAGAGAGTGAGGCATTGGTCT	
IGF2-FW	GCTGACCTCATTTCCCGATAC	
IGF2-REV	GGCGTCTCTTCTGCTTCTTT	
Camk1g-FW	GCATCGTCCACAGAGATCTAAA	
Camk1g-REV	ACTCCATTCTGCTCCATCTTG	
TUT7-FW	ACCTGTGTTTCAGACCCTTAC	
TUT7-REV	CAGCTGCATTTCCCAGTTTATC	
TUT4-FW	GAAGACAGAAACAGACAACCA	
TUT4-REV	CCTCCAAAGCAAAATCCAGTG	
b-Actin-FW	CCAACCGTGAAAAGATGACC	
b-Actin-REV	CATCACAATGCCTGTGGTAC	
7SL-FW4-mouse_specific	CCAGCTACTCGGGAGGCTGAGACA	
7SL-REV1-mouse_specific	CTGATCAGCACGGGAGTTT	
7SL-REV3-mouse_specific	TCACCATATTGATGCCGAACTTA	This oligo was used for northern blot analysis of 7SL RNA
**cRACE**		
7SL-cRACE-FW	GACCACCAGGTTGCCTAAG	
7SL-cRACE-REV	CTGATCAGCACGGGAGTTT	
**Cloning 7SL locus into pGEM-T easy vector**		
7SL-FW	GGTAGTAATTTAAAGAGTGAGGGATAGAGAGCC	
7SL-REV	TACCCACGGACTAGCTTTCCTCC	
**Cloning 7SL locus into pLKO.1 vector**		
SalI_7SL-Fw	CCCCTTCACCGTCGACTACCCACGGACTAGCTTTCC	
EcoRI_7SL_Trunc-REV	TCGAGAATTCACGCGTGCAAAATGGAGTGCAGTGGCTATTCACAG	used for cloning truncated-7SL RNA
**Sub-cloning Renila luciferase into U6 promoter-less pLKO.1 vector**		
	Features: NheI -Insulin Signal Peptide Sequence- 3 aa linker - 5'start of the hRluc	
Insulin_hRluc_F_NheI	CTATAGGCTAGCCACCatggcgctgtggatgcgcctgctgccgctgctggcgctgctggcgctgtggggcccggatccggcggcggcgGACCGGTCGATGGCTTCCAAGGTGTACGAC	This oligo was used to insert signal peptide encoding sequence into the 5'-end of the Renila luciferase
	Features: 3'_end of hRluc - KDEL - Stop - XhoI	
hRluc_KDEL_R_XhoI	GGCTCGAGCGATCGCCTAGAATTACAGCTCGTCCTTCTGCTCGTTCTTCAGCAC	This oligo was used to insert ER retention coding (KDEL) encoding sequence into the 3'-end of the Renila luciferase
hRluc_R_XhoI	GGCTCGAGCGATCGCCTAGAATTACTGCTCGTTCTTCAGCAC	This oligo was used to amplify Renila luciferase without the insertion of the ER retention coding (KDEL) encoding sequence
