# Supplementary material for: The Perlman syndrome DIS3L2 exoribonuclease safeguards endoplasmic reticulum-targeted mRNA translation and calcium ion homeostasis
Source: Nat Commun. 2020 May 26;11:2619. doi: 10.1038/s41467-020-16418-y (PMC7250864; doi:10.1038/s41467-020-16418-y)
Supplement: Supplementary file 3 — Reporting Summary [file 41467_2020_16418_MOESM3_ESM.pdf]

nature research

Corresponding author(s): Richard I. Gregory

Last updated by author(s): Apr 5, 2020

# Reporting Summary

Nature Research wishes to improve the reproducibility of the work that we publish. This form provides structure for consistency and transparency in reporting. For further information on Nature Research policies, see Authors & Referees and the Editorial Policy Checklist.

## Statistics

For all statistical analyses, confirm that the following items are present in the figure legend, table legend, main text, or Methods section.

| n/a | Confirmed | |
|---|---|---|
| ☐ | ☒ | The exact sample size (*n*) for each experimental group/condition, given as a discrete number and unit of measurement |
| ☐ | ☒ | A statement on whether measurements were taken from distinct samples or whether the same sample was measured repeatedly |
| ☐ | ☒ | The statistical test(s) used AND whether they are one- or two-sided<br>*Only common tests should be described solely by name; describe more complex techniques in the Methods section.* |
| ☒ | ☐ | A description of all covariates tested |
| ☒ | ☐ | A description of any assumptions or corrections, such as tests of normality and adjustment for multiple comparisons |
| ☐ | ☒ | A full description of the statistical parameters including central tendency (e.g. means) or other basic estimates (e.g. regression coefficient) AND variation (e.g. standard deviation) or associated estimates of uncertainty (e.g. confidence intervals) |
| ☐ | ☒ | For null hypothesis testing, the test statistic (e.g. *F*, *t*, *r*) with confidence intervals, effect sizes, degrees of freedom and *P* value noted<br>*Give P values as exact values whenever suitable.* |
| ☒ | ☐ | For Bayesian analysis, information on the choice of priors and Markov chain Monte Carlo settings |
| ☒ | ☐ | For hierarchical and complex designs, identification of the appropriate level for tests and full reporting of outcomes |
| ☒ | ☐ | Estimates of effect sizes (e.g. Cohen's *d*, Pearson's *r*), indicating how they were calculated |

*Our web collection on statistics for biologists contains articles on many of the points above.*

## Software and code

Policy information about availability of computer code

| | |
|---|---|
| Data collection | *Provide a description of all commercial, open source and custom code used to collect the data in this study, specifying the version used OR state that no software was used.* |
| Data analysis | Subread v1.6.3 and featureCounts v1.6.3 were used to analyze RNA-sequencing data. For the Tail-sequencing data analysis R v3.6 and RStudio v1.2.1335 were used. The detailed information about this tool and home-made codes are published elsewhere and cited in this manuscript (Pirouz et. al., METHODS, 2019) and a brief description is provided in the Method section. "Prism-GraphPad" was used for statistical analysis as well as data depiction. |

For manuscripts utilizing custom algorithms or software that are central to the research but not yet described in published literature, software must be made available to editors/reviewers. We strongly encourage code deposition in a community repository (e.g. GitHub). See the Nature Research guidelines for submitting code & software for further information.

## Data

Policy information about availability of data

All manuscripts must include a data availability statement. This statement should provide the following information, where applicable:

- Accession codes, unique identifiers, or web links for publicly available datasets
- A list of figures that have associated raw data
- A description of any restrictions on data availability

RiboSeq data (GSE136350) and RNA-Sequencing data (136334) are deposited in GEO and are publicly available

# Field-specific reporting

Please select the one below that is the best fit for your research. If you are not sure, read the appropriate sections before making your selection.

☒ Life sciences ☐ Behavioural & social sciences ☐ Ecological, evolutionary & environmental sciences

For a reference copy of the document with all sections, see nature.com/documents/nr-reporting-summary-flat.pdf

# Life sciences study design

All studies must disclose on these points even when the disclosure is negative.

| | |
|---|---|
| Sample size | No statistical model was used to predetermine sample size. For any in vitro experiment, "n" was chosen based on the previous publications in the field: at least 3 independent biological in vitro samples were tested. For qRT-PCR experiments, at least 3 technical replicates were analyzed. |
| Data exclusions | No specific data was excluded. |
| Replication | In the Figure legend, we stated how many number of replications were performed. We also stated P-value to show the statistical significance. |
| Randomization | Experiments were not randomized. Cell cultures were randomly assigned to different groups or treatments. |
| Blinding | The experiments were not blinded. |

# Reporting for specific materials, systems and methods

We require information from authors about some types of materials, experimental systems and methods used in many studies. Here, indicate whether each material, system or method listed is relevant to your study. If you are not sure if a list item applies to your research, read the appropriate section before selecting a response.

## Materials & experimental systems

| n/a | Involved in the study |
|---|---|
| ☐ | ☒ Antibodies |
| ☐ | ☒ Eukaryotic cell lines |
| ☒ | ☐ Palaeontology |
| ☒ | ☐ Animals and other organisms |
| ☒ | ☐ Human research participants |
| ☒ | ☐ Clinical data |

## Methods

| n/a | Involved in the study |
|---|---|
| ☒ | ☐ ChIP-seq |
| ☐ | ☒ Flow cytometry |
| ☒ | ☐ MRI-based neuroimaging |

## Antibodies

| | |
|---|---|
| Antibodies used | These antibodies were used for western blotting: anti-FLAG® M2-Peroxidase (HRP) (Sigma; A8592, 1:10000 dilution), anti-DIS3L2 (Novusbio; NBP1-84740, 1:1000 dilution), anti-b-ACTIN (Abcam; ab8227, 1:5000 dilution), anti-SRP68 (Proteintech Group; 11585-1-AP, 1:1000 dilution), anti-RPL23a (Proteintech Group; 16386-1-AP, 1:1000 dilution), anti-SIX2 (Abcam; ab111827, 1:500 dilution), anti-PAX2 (a kind gift from Dr. Kreidberg; 1:1000 dilution), anti-FGF9 (Abcam; ab206408, 1:1000 dilution).<br>For immunoprecipitation, FLAG-M2-magnetic beads (M8823, Sigma), anti-RPL23a (Proteintech Group; 16386-1-AP), or normal rabbit IgG (Cell Signaling, 2729) were used. |
| Validation | Anti-FLAG® M2-Peroxidase (HRP) (Sigma; A8592): validated on recombinant FLAG peptide by Sigma, and widely used in the field.<br>Anti-DIS3L2 (Novusbio; NBP1-84740): validated in-house using DIS3L2 wildtype and knockout cells as positive and negative controls, respectively.<br>Anti-b-ACTIN (Abcam; ab8227): validated in western blot analysis of extract from mouse brain tissue lysate, HeLa cells, etc. by Abcam.<br>Anti-SRP68 (Proteintech Group; 11585-1-AP): validated in western blot analysis of extract from mouse brain tissue by Proteintech Group.<br>Anti-RPL23a (Proteintech Group; 16386-1-AP): validated in western blot analysis of extract from HepG2 cells by Proteintech Group.<br>Anti-SIX2 (Abcam; ab111827): validated in western blot analysis of extracts from human, mouse and rat skeletal muscle tissues by Abcam.<br>Anti-PAX2 (a kind gift from Dr. Kreidberg): Generated in-house and validated in western blot analysis of mouse kidney tissue lysate.<br>Anti-FGF9 (Abcam; ab206408): validated in western blot analysis of recombinant human FGF9 protein by Abcam. |

# Eukaryotic cell lines

Policy information about cell lines

| | |
|---|---|
| Cell line source(s) | mouse embryonic stem cells (TC1) line, a gift from Dr. Alt.<br>Human cancer cell line HEK 293T was purchased from ATCC.<br>Human cancer HCT116 line was purchased from ATCC.<br>Human skin fibroblast BJ line was purchased from ATCC. |
| Authentication | TC1 mouse ESC line was authenticated in-house by multi-lineage differentiation and expression of pluripotency markers.<br>HEK293T, HCT116, and BJ lines have been authenticated by ATCC. |
| Mycoplasma contamination | All the cell lines were tested negative for mycoplasma contamination. |
| Commonly misidentified lines<br>(See ICLAC register) | No commonly misidentified cell line was used. |

# Flow Cytometry

## Plots

Confirm that:

☒ The axis labels state the marker and fluorochrome used (e.g. CD4-FITC).

☒ The axis scales are clearly visible. Include numbers along axes only for bottom left plot of group (a 'group' is an analysis of identical markers).

☒ All plots are contour plots with outliers or pseudocolor plots.

☒ A numerical value for number of cells or percentage (with statistics) is provided.

## Methodology

| | |
|---|---|
| Sample preparation | 8.5 days after renal differentiation of Osr1-GFP cells (at metanephric mesenchyme stage), cell aggregates were dissociated with type 1 Collagenase 1.5 mg/mL (Worthington Biochemical) for 10 minutes at room temperature with gentle shaking. Dissociated cells were centrifuges, washed with PBS (Invitrogen) and resuspended in 500 ml PBS and analyzed by flowcytometry (BD FACSAria). Undifferentiated mESCs (not-expressing GFP) were used as the negative control. |
| Instrument | BD FACSAria |
| Software | Data were collected using DIVA (Becton Dickinson) software and analyzed using FlowJo software (Tree Star, Inc.). |
| Cell population abundance | No post-sorting analysis was done. |
| Gating strategy | Debris and dead cells were excluded by forward and side scatter gating. Gating for GFP positive and negative populations was selected using undifferentiated Osr1-GFP mESCs as negative controls (Extended Data Figure 7) |

☒ Tick this box to confirm that a figure exemplifying the gating strategy is provided in the Supplementary Information.
