# Supplementary material for: The Perlman syndrome DIS3L2 exoribonuclease safeguards endoplasmic reticulum-targeted mRNA translation and calcium ion homeostasis
Source: Nat Commun. 2020 May 26;11:2619. doi: 10.1038/s41467-020-16418-y (PMC7250864; doi:10.1038/s41467-020-16418-y)
Supplement: Supplementary file 4 — Description of Additional Supplementary Files [file 41467_2020_16418_MOESM4_ESM.pdf]

Description of Additional Supplementary Files

File Name: Supplementary Data 1
Description: Ribosome profiling analysis of DIS3L2 knockout mESCs.

File Name: Supplementary Data 2
Description: RNA sequencing analysis of gene expression in shLacZ and shDIS3L2 ESCs during differentiation.

File Name: Supplementary Data 3
Description: RNA sequencing analysis of selected genes expression in shLacZ and shDIS3L2 ESCs during differentiation with respective fold changes.
